# Virtual patients versus standardized patients for improving clinical reasoning skills in ophthalmology residents. A randomized controlled trial

**DOI:** 10.1186/s12909-024-05241-4

**Published:** 2024-04-22

**Authors:** Tayyaba Gul Malik, Usman Mahboob, Rehan Ahmed Khan, Rabail Alam

**Affiliations:** 1 Masters in Medical Education (Scholar), University of Lahore, Lahore, Pakistan; 2 Institute of Health Professions Education & Research, Khyber Medical University, Peshawar, Pakistan; 3 Dean Riphah Institute of Assessment, Riphah International University, Islamabad, Pakistan; 4 IMBB, University of Lahore, Lahore, Punjab Pakistan

**Keywords:** Virtual patients, Standardized patients, Ophthalmology, Clinical reasoning, Pretest, Posttest

## Abstract

**Background:**

History taking and clinical reasoning are important skills that require knowledge, cognition and meta-cognition. It is important that a trainee must experience multiple encounters with different patients to practice these skills. However, patient safety is also important, and trainees are not allowed to handle critically ill patients. To address this issue, a randomized controlled trial was conducted to determine the effectiveness of using Virtual Patients (VP) versus Standardized Patients (SP) in acquiring clinical reasoning skills in ophthalmology postgraduate residents.

**Methods:**

Postgraduate residents from two hospitals in Lahore, Pakistan, were randomized to either the VP group or the SP group and were exposed to clinical reasoning exercise via the VP or SP for 30 min after the pretest. This was followed by a posttest. One month after this activity, a follow-up posttest was conducted. The data were collected and analysed using IBM-SPSS version 25. Repeated measures ANOVA was used to track the effect of learning skills over time.

**Results:**

The mean age of the residents was 28.5 ± 3 years. The male to female ratio was 1:1.1. For the SP group, the mean scores were 12.6 ± 3.08, 16.39 ± 3.01 and 15.39 ± 2.95, and for the VP group, the mean scores were 12.7 ± 3.84, 16.30 ± 3.19 and 15.65 ± 3.18 for the pretest, posttest and follow-up posttest, respectively (p value < 0.00). However, the difference between the VP and SP groups was not statistically significant (*p* = 0.896). Moreover, there was no statistically significant difference between the VP and SP groups regarding the retention of clinical reasoning ability. In terms of learning gain, compared with the VP group, the SP group had a score of 51.46% immediately after clinical reasoning exercise as compared to VP group, in which it was 49.1%. After one month, it was 38.01 in SP and 40.12% in VP group.

**Conclusion:**

VPs can be used for learning clinical reasoning skills in postgraduate ophthalmology residents in a safe environment. These devices can be used repeatedly without any risk to the real patient. Although similarly useful, SP is limited by its nonavailability for repeated exercises.

**Supplementary Information:**

The online version contains supplementary material available at 10.1186/s12909-024-05241-4.

## Background

History taking and clinical reasoning are important skills that require knowledge, cognition and meta-cognition. These tools, if properly used, help in the diagnosis and management of patients. It is important that a trainee must experience multiple encounters with different patients. However, health authorities are very sensitive to patient safety, and trainees are not allowed to handle critically ill patients. This creates a problem in the training of residents, and direct patient encounters with residents are becoming difficult each year. Although mannequin-based simulations have been used to address these issues, these are very expensive and lack interpersonal communication. This can be overcome by using digital or computer-based simulations called virtual patients (VPs).

VP is essentially a computer program in which real-life scenarios are presented as digital patients, and the trainee takes up the role of a doctor, takes history and makes decisions regarding diagnosis and management [1]. The idea in using a VP is that the user is free to make decisions based on the information provided by the VP. The clinician should make differential diagnoses and develop treatment plans for patients in a safe environment. Thus, the VP can be used as a good tool for assessing clinical reasoning skills [2]. 

Although we found research on developing clinical reasoning skills using VPs, the results of medicine and surgery cannot be generalized to ophthalmology due to the differences in examination techniques/tools, clinical presentations, investigations and treatments used. In ophthalmology; Slit Lamp Bio-microscopy, visual acuity testing, Tonometry and fundus examination are some of the techniques, which are entirely different from those used in other specialties [3, 4]. In ophthalmology, we find the use of simulations to help patients with low vision, to treat amblyopia, to acquire practical skills, to practice cover-un-cover tests, to perform ophthalmoscopy and to perform surgeries [5]. However, studies comparing the use of VPs and standardized patients (SPs) for developing clinical reasoning skills in Ophthalmology is scarce.

This randomized controlled trial was conducted to determine the effectiveness of using the VP (as an educational intervention) on the clinical reasoning skills of ophthalmology residents, as measured by pretest and posttest scores. The scores were compared between the intervention group and the control group (using standardized patients). The secondary objective was to evaluate the retention of clinical reasoning by repeating the posttest after one month.

## Methods

This was a randomized controlled trial conducted at two tertiary care teaching hospitals affiliated with the Post Graduate Medical Institute/Lahore General Hospital (PGMI/LGH), Lahore Hospital and the College of Ophthalmology and Allied Vision Sciences/Mayo Hospital (COAVS/Mayo Hospital), Lahore, Pakistan. The study was approved by the institutional review board (Ref: ERC lQ3l23l0l). The study was conducted in accordance with the Declaration of Helsinki. Using Open-Epi sample size calculator with a 5% margin of error and 80% power, the sample size was 38 (taking percentage of exposed with outcome as 50). Total number of residents in these two hospitals were 60 at the time of study. Ten were in the first three months of their training and did not qualify for inclusion. However, we included 50 residents, 3 dropped out and 47 were included in study. Postgraduate residents of LGH and Mayo Hospital, Lahore, who consented to be part of the study, were included and randomized by an Excel random generator to either the VP group or the SP group. Consent was taken from all residents for both study participation and publication of information in an online open-access publication.

Residents of all four training years, either by sex or age > 25 years, were included, and residents who were recently inducted with less than 3 months of training were excluded. There were 50 residents who consented to participate from two institutions (LGH = 20 and Mayo = 30). Three residents from LGH later on declined, and forty-seven were included, keeping in view of the possibility of further dropout. Participants with VPs were classified as the intervention arm, and the participants who were allocated to the SP arm were classified as the control arm. There were 24 participants in the intervention group and 23 in the control group.

**Fig. 1:**
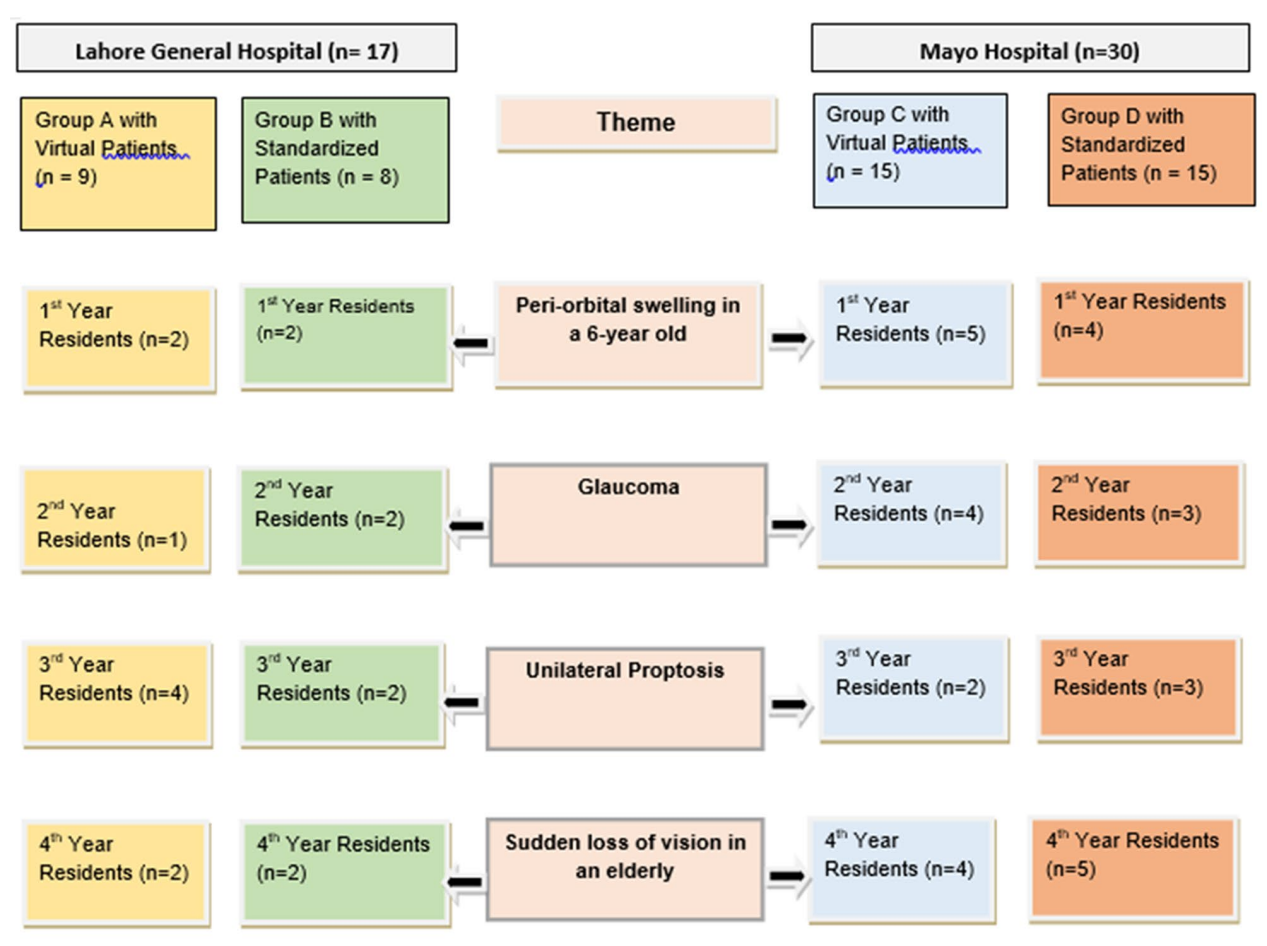
Flow chart 1, showing the distribution of patients according to year of residency

Participants in the intervention arm were exposed to clinical reasoning exercise via the VP. Similar patients were presented to the intervention and control arms of the same year of residency. However, depending on the case difficulty, residents were provided with different cases in different years. The details of the group distribution and patient allocation are shown in Fig. 1 (flow chart 1).

VPs were selected from the ‘American Academy of Ophthalmology’ website (https://www.aao.org/cme-central). A formal email was sent to obtain permission from the Academy to use the VP for research purposes, which was granted. The VPs were clinical scenarios on the computer screen.

After the patient’s medical history and ocular examination, relevant investigations were performed to reach a diagnosis. The residents had to choose relevant investigations based on the information provided. The final diagnosis was obtained from the resident, and the case ended if the answer was correct. The resident could take another chance if the answer was incorrect. The whole process lasted 30 to 45 min.

**Fig. 2:**
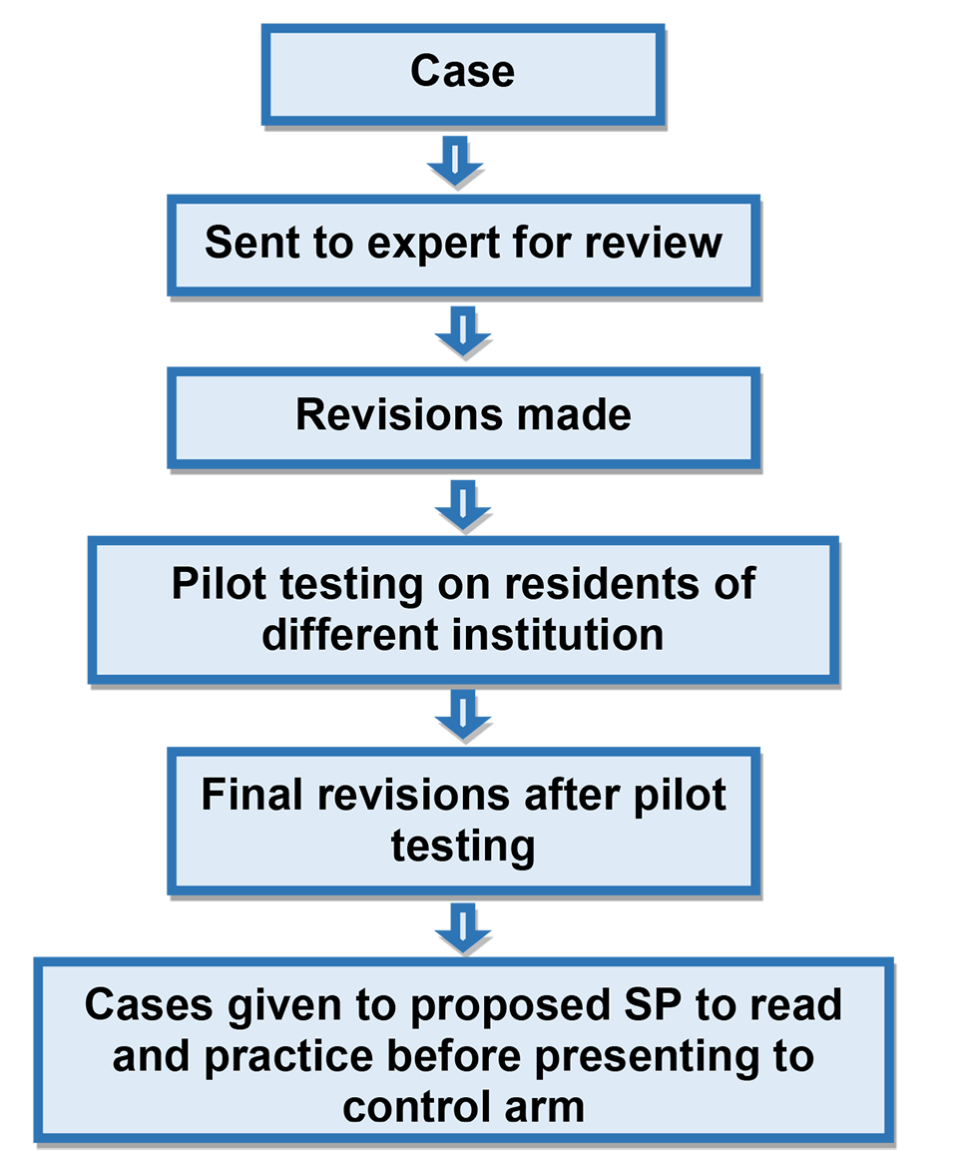
Flow chart 2 showing the development of standardized patients in the control arm

Four SPs were developed using similar cases. The SPs were reviewed and validated by five ophthalmologists with at least five years of experience in training postgraduate residents. The cases were pilot tested on four residents of four training years at a hospital other than the institute included in this research, and their opinions were incorporated into the case scenarios. Informed consent was obtained from the SP before roleplay was allocated. SPs were given instructions regarding their disease. History, clinical examination findings and important investigations were provided to the SPs. Figure 2 and flow chart 2 show the development of SPs.

**Fig. 3:**
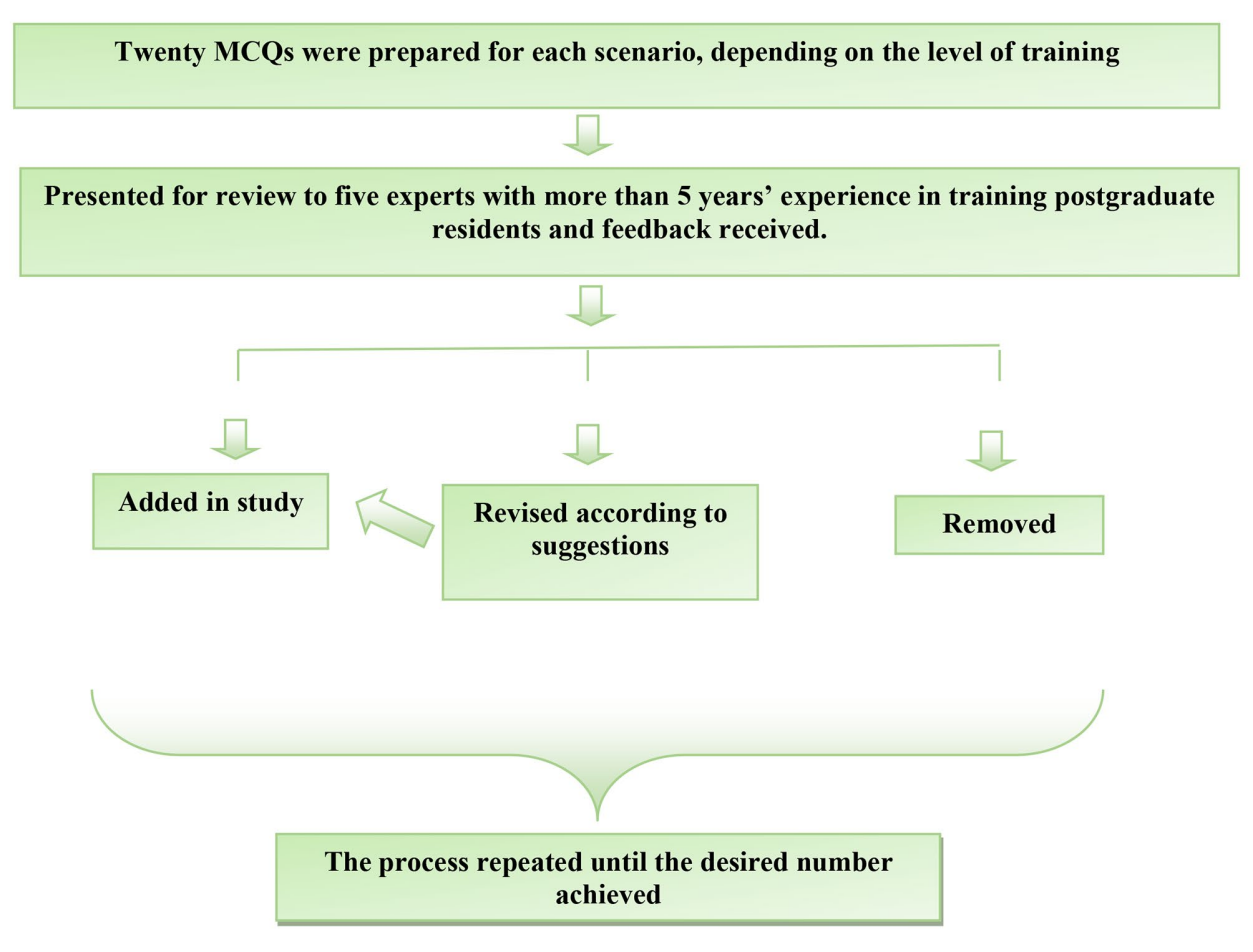
Flowchart 3 for preparation of multiple choice questions

Twenty multiple-choice questions (MCQs) related to the case scenarios were prepared for each case, which were the same for both groups. MCQs were reviewed and validated by experts with more than five years of experience in training residents. Each pretest and posttest consisted of 10 MCQs selected from the 20 MCQs for each scenario. Each MCQ carried one mark. Figure 3 with flow chart 3 shows the construction of the MCQs.

The residents who consented to participate were given the details and purpose of the study and the time, venue and steps involved (pretest, clinical reasoning exercise, posttest and follow-up posttest).

On the day of activity, an MS-EXCEL random number generator was used to assign VPs and SPs to the participants. After briefing, the pretest was presented to all participants according to their year of residency. The results of the pretest were taken as a baseline score for each student. The pre- and posttest anonymity was ensured by the use of a colour name followed by the last 4 digits of the phone numbers. This number was used in both the pretest, posttest and follow-up posttests. VP and SP were given to the corresponding group for a period of 30 min, which could be extended to 45 min if the resident required additional time. This was followed by a posttest.

**Fig. 4:**
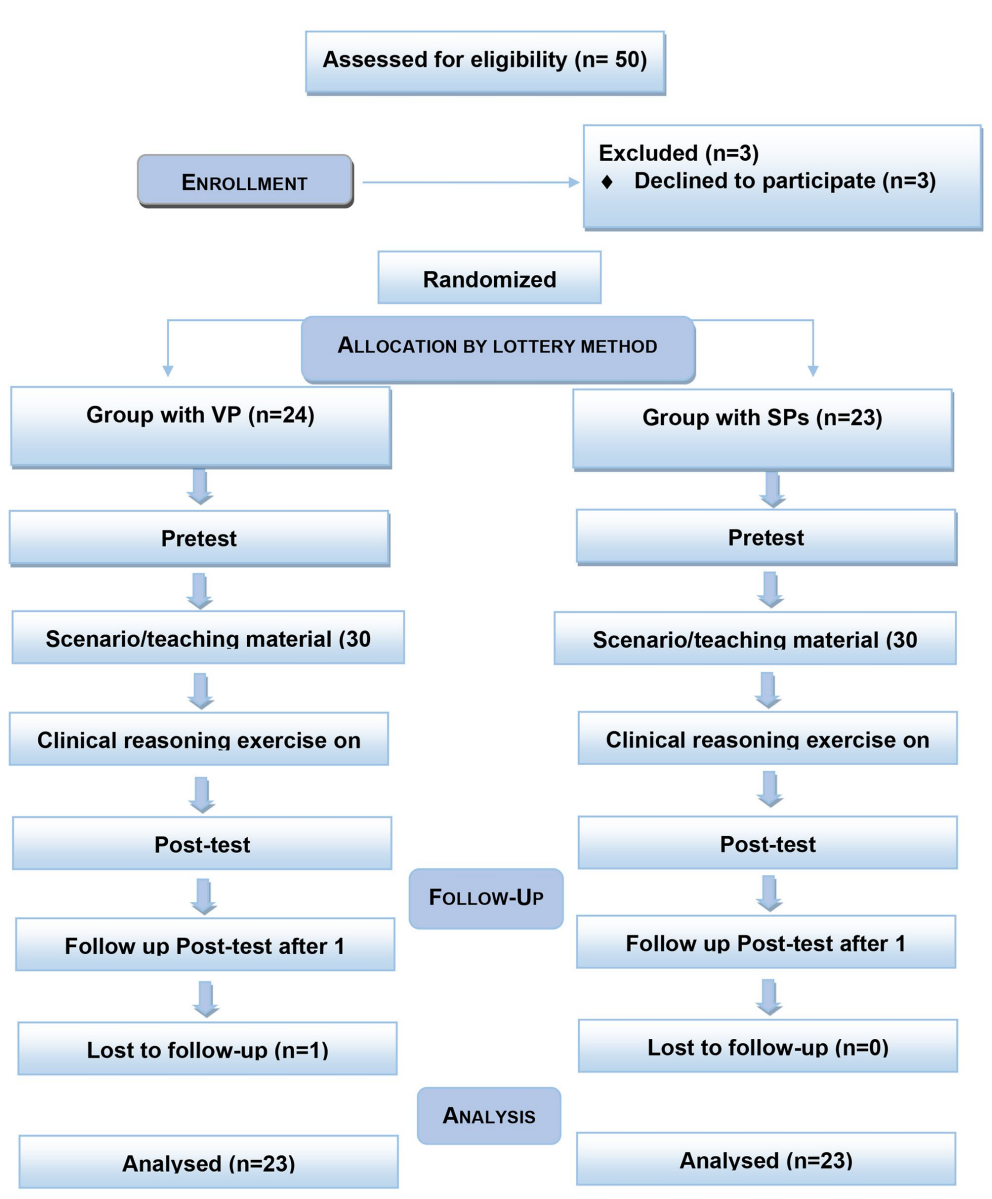
Flow chart 4 showing the CONSORT guidelines for reporting the trial

One month after this educational activity, the participants were informed that the groups would appear in the follow-up posttest. The data were collected in Excel files and analysed using ‘IBM-SPSS’ version 25. For categorical variables, descriptive statistics are presented as the frequency and percentage. Repeated measures ANOVA was used to track the effect of learning skills over time. For pairwise comparisons, the Bonferroni test was used. CONSORT guidelines were followed for reporting the data (Fig. 4 with flow chart 4).

## Results

Of the 47 participants, one resident did not appear in the follow-up posttest and was excluded from the final analysis. The mean age of the residents was 28.5 ± 3 years. The male-to-female ratio was 1:1.1 (24 females and 22 males).

The mean pretest score for the VP was 12.7 ± 3.84, which improved to 16.30 ± 3.19 in post-test with p value of < 0.00. Clinical reasoning skills significantly improved with the use of the VP. For the SP group, the mean scores were 12.6 ± 3.08, 16.39 ± 3.01 and 15.39 ± 2.95 for the pretest, posttest and follow-up posttest, respectively. For the VP group, the mean scores were 12.7 ± 3.84, 16.30 ± 3.19 and 15.65 ± 3.18 for the pretest, posttest and follow-up posttest, respectively (Tables 1 and 2). Although there were significant differences among the pretest, posttest and follow-up posttest scores in the VP and SP groups (p value < 0.00), the difference between the VP and SP groups was not statistically significant (*p* = 0.896), indicating that both techniques were equally effective at acquiring clinical reasoning skills in ophthalmology residents.

**Table 1:** Mean pretest, posttest and follow-up values between SPs and VPs according to year of residency

Years of residency	SP group			VP group		
	Pre-test	Post-test	Follow up post-test	Pre-test	Post-test	Follow up post-test
1st year	13.33 ± 2.7	17 ± 2.4	15.7 ± 2.7	13.5 ± 2.07	17.2 ± 2.2	16 ± 2.1
2nd year	11.4 ± 3.8	15.4 ± 4.7	15.2 ± 3.8	8.6 ± 4.7	15.4 ± 4.7	11.8 ± 3.9
3rd year	14.4 ± 1.9	17.2 ± 1.8	15.8 ± 1.8	15.5 ± 2.7	18.5 ± 0.5	17.5 ± 1.0
4th year	11.4 ± 3.3	16 ± 3.1	15 ± 3.7	12.7 ± 2.9	16.8 ± 1.7	16.7 ± 2.5

**Table 2:** Analysis using repeated-measures ANOVA showing differences in p values

Analysis by using repeated measure ANOVA				
Training year of residents			Pairwise comparison by Bonferroni correction	
	SP vs. VP (P value)	Pre-test vs. Post-test vs. Follow-up Post-test (P value)	Pre-test vs. Post-test (P value)	Pre-test vs. Follow-up Post-test (P value)
1st year	0.848	0.014	0.002	0.041
2nd year	0.231	0.005	0.01	0.016
3rd year	0.171	0.01	0.002	0.031
4th year	0.424	0.001	< 0.00	0.002
All residents	0.896	< 0.00	< 0.00	< 0.00

In the follow -up posttest, there was a slight decrease in the scores compared to those of the posttest, ranging from − 0.1 to − 1.4. However, the net gain in clinical reasoning skill significantly improved from the pretest to the follow-up posttest (p value < 0.00). There was no statistically significant difference between the VP and SP groups regarding retention of clinical reasoning ability or net gain in clinical reasoning skill from pretest to follow-up posttest.

To calculate learning gain, we applied calculations used by Barwood et al. [6] as follows:

(*Total Posttest Score obtained - **Total Pretest Score obtained) × 100.

(***Sum of Maximum Score - Total Pre − test Score obtained)

* Sum of individual posttest scores of all the participants

** Sum of individual pretest scores of all the participants

*** sum of all the scores, i.e., 20 × 28 = 560

**Graph 1:**
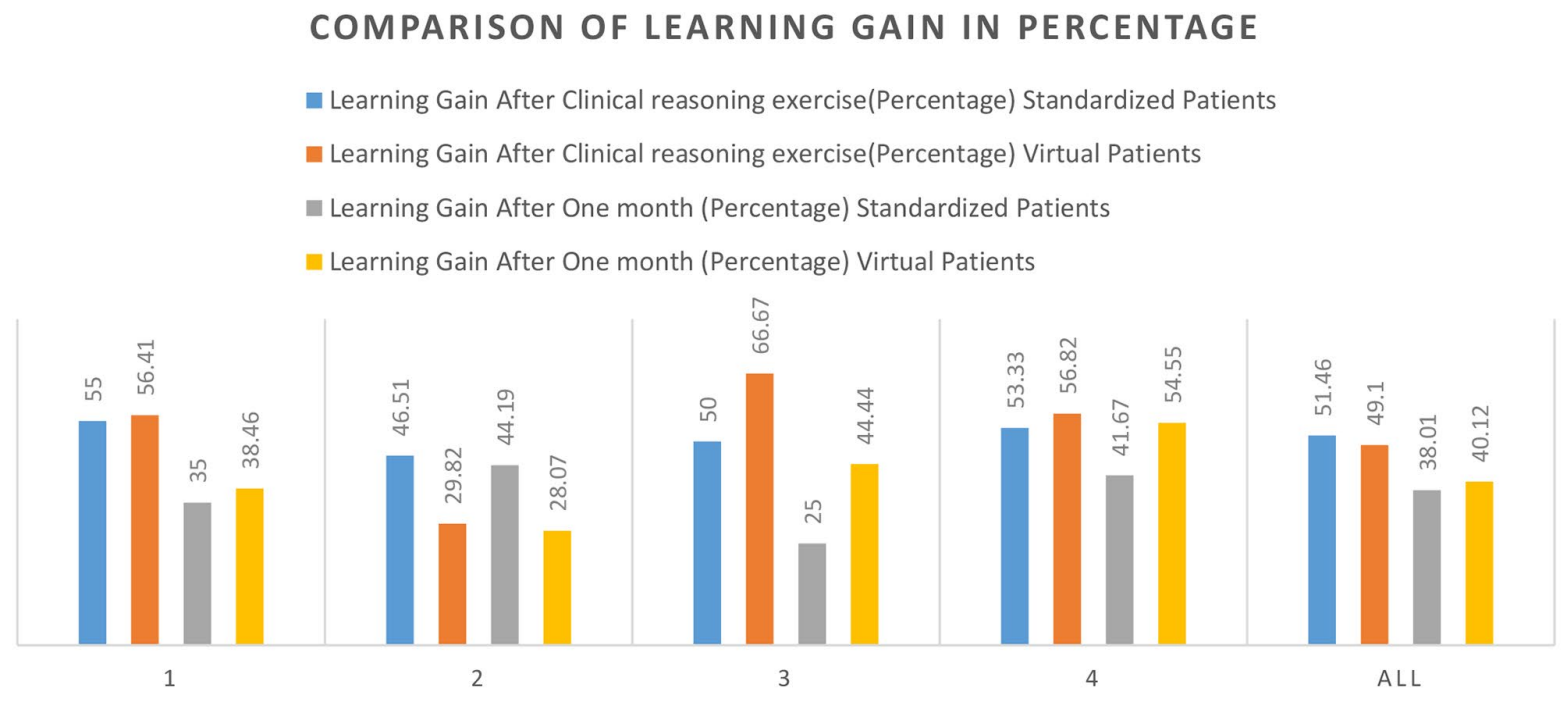
Comparison of learning gains in different years of residency. *‘1, 2, 3, 4’ indicate year of residency, and ‘All’ indicates all residents*

In the SP group, there was a net learning gain of 51.46% immediately after clinical reasoning exercise compared to that in the VP group, in which it was 49.1%. After one month, it was 38.01% in the SP group and 40.12% in the VP group (Graph 1).

## Discussion

The results of this study showed that clinical reasoning skills significantly improved with the use of VPs and SPs (*p <* 0.00). However, the difference between the final results of the VP and SP groups was not statistically significant (*p* = 0.896), indicating that both techniques were equally effective at acquiring clinical reasoning skills in ophthalmology residents. In contrast to our results, a study from Sydney in a Virtual Ophthalmology Clinic rotation with VP showed a statistically significant improvement in the experimental group compared to the control group [7]. The difference in the results could be because of the use of knowledge-based tests in the Sydney study compared to our focus on clinical reasoning skills. They used traditional teaching as a control method with crossover trials and conducted studies on undergraduate students, which were dissimilarities from our study. The Sydney trial was the only study in ophthalmology and was very close to our study design. As VPs are available and accessible at any time of day and one can learn at one’s own pace in a safe and controlled environment, much work needs to be done in Ophthalmology, for which little evidence is available.

Among other specialties, in the field of internal medicine, in contrast to the results of the Sydney trial, there was no superiority of VP over traditional methods. However, there was better retention of knowledge associated with VPs than with traditional methods [8]. This was different from our results as well.

During the COVID-19 era, many trials were conducted in different medical specialities, which supported the effectiveness and value of VPs in medical education, especially in providing a safe learning environment. In Brazil, a quasi-experimental study with pre and post intervention assessment was carried out using a mobile application for virtual clinical simulation [9]. The experimental group had a higher final score than the control group.

In a Neurology course, 117 students were enrolled to improve interviewing and diagnostic skills for cranial nerve disorders [10]. Although the assessment scores after using the VP were low, the students learned from their mistakes and received feedback from the system while continuing to exercise. The author highlighted the importance of virtual simulation, as learners can learn from mistakes, which is not possible in real patients. Thus, VPs are useful tools for learning.

In managing oncological emergencies, pilot testing of VPs was performed to improve learner skills [2]. There was an increase in the mean test result from 58 to 86% in the pretest and posttest, respectively. In our VP group, the mean percentage of pretest scores was 62.8%, which improved to 81.96% in the posttest. The percentage of improvement in our participants was very close to that reported by Fawaz et al. Both studies included postgraduate residents.

In a surgical clerkship involving two different diseases treated with the VP, the importance of the VP in terms of repetition has been highlighted, but this approach is not easy to apply in human simulations [11]. 

In Family Medicine, a case‒controlled study revealed no statistically significant difference between the VP group and the control group [12]. However, a statistically significant difference was observed between the initial and final knowledge of both groups. The control in this study was paper-based scenarios, in contrast to our standardized patients. However, the results were similar in terms of the pretest and posttest scores.

The local literature from Pakistan shows a scarcity of data regarding the VP. In a study at Dow University of Health Sciences, Karachi, Pakistan, paper-based simulations were compared with computer-based simulations using Articulate Storyline software [13]. In contrast to our study, which used MCQs, objective structured clinical examination (OSCE) was used to assess clinical reasoning skills. The results showed the superiority of the VP in paper-based scenarios.

Several studies have highlighted different aspects of the VP. Torres et al. noted the importance of VPs in improving decision-making with repeated exercises, which is ethically not possible for real patients and difficult for standardized patients [14]. Other authors have compared different approaches using VPs [15, 16]. Many types of online software are available for developing clinical reasoning skills using VPs. We used AAO for our study. Another tool for VP is eCREST—‘the electronic Clinical Reasoning Educational Simulation Tool’. An RCT was carried out using eCREST, which showed improved clinical reasoning with the eCREST compared to that of the control group [17]. It is important to emphasize that VPs are specialty specific. The way they are prepared and the software used for construction are also important.

Evidence from review articles showed that the earliest review article in our selected literature (from 2009 to 2023) was by Cook et al. [1]. Another review article showed that 86% of studies supported virtual simulation as an effective learning strategy [18]. According to a review of 12 studies, online virtual simulation was found to be comparable or superior to other traditional methods [19]. However, this review did not include ophthalmology.

In a meta-analysis, VPs were compared with traditional methods, blended education, types of digital education, and different design variants of VPs in health profession education [20]. The analysis showed similar results for knowledge gained through VPs and traditional education. However, the results favoured the use of VPs for skills development. This review is important because it provides evidence from both high-income and low- and middle-income countries. A literature review also revealed that much of the related work on VPs involved nurses [21, 22]. Hence, further evidence is needed for undergraduate and postgraduate learners.

As the use of the VP and its importance in medical education is evolving, we found different suggestions and recommendations for the use of the VP in medical education. Baumann-Birkbeck et al. suggested that the blended-learning (BL) approach might be more beneficial due to individual learning styles [23]. Similarly, Plackett et al. reported that VPs might effectively complement traditional teaching [24]. 

With this mixed nature of evidence from different studies and different fields of medicine, this particular RCT, which was conducted in the field of Ophthalmology from two different tertiary care centres in Lahore, Pakistan, will add up to the literature and will help in creating further evidence in this field, especially from low- to middle-income countries. The clinical medical education systems of our country is quite different from high-income countries. This study provides evidence for the effective use of VPs in developing countries.

A limitation of this study is that only public sector institutes from Lahore city were included in this trial, and data from the private sector are lacking; additionally, these institutions have different dynamics and include fewer patients. We included only one month of follow-up, which can be extended to one year for future research. Only one case scenario was given to each resident according to the difficulty. More cases can provide further concrete evidence. Moreover, Bed side manners, patient welfare, communication skills cannot be taught by VP.

Future research questions can be developed regarding time and place of using VP (for example, what is the limit up to which VPs can be blended in traditional education?). Comparisons between different types of VP are another area to be explored. A temporal comparison between the VP and SP activities can also be performed. A comparison between private and public sector institutions regarding perception and acceptability can also be put into a research question. Research involving different years of residency can be conducted to determine whether VPs are more useful for the early or later years of residency programs.

### Electronic supplementary material

Below is the link to the electronic supplementary material.

Supplementary Material 1

## Data Availability

Data is provided as supplementary information files. Standardized patient instructions, and the reading material provided to the residents are given in the supplementary file.

## List of abbreviations

VPs: Virtual patients
SPs: Standardized patients
PGMI: Postgraduate Medical Institute
LGH: Lahore General Hospital
COAVS: College of Ophthalmology and Allied Vision Sciences
MCQs: Multiple-choice questions
OSCE: Objective structured clinical examination
BL: Blended learning
AAO: American Academy of Ophthalmology
