# Supplementary material for: Virtual patients versus standardized patients for improving clinical reasoning skills in ophthalmology residents. A randomized controlled trial
Source: BMC Med Educ. 2024 Apr 22;24:429. doi: 10.1186/s12909-024-05241-4 (PMC11034047; doi:10.1186/s12909-024-05241-4)
Supplement: Supplementary file 1 — Supplementary Material 1 [file 12909_2024_5241_MOESM1_ESM.pdf]

SIMULATED PATIENT FOR 1ST YEAR RESIDENT

Instruction to the patient

During the Interview

- The interview will last approximately 15-30 minutes. Try to remain focused on the role you are playing. If possible, do not keep the written role in front of you during the interview.
- You can assist the resident by revealing some information as appropriate.
- Act naturally and react as though you are the actual patient you are portraying.

Behaviors to exhibit

- You are cooperative
- You speak Urdu well, but ask for clarification frequently, especially if medical jargon is used
- You are very concerned about seriousness of the lid swelling and also concerned that the child does not allow to touch the tender lid.
- You have no problems taking the medications

Patient details

Setting

You are mother/father of a 6-year-old girl who visits out patient department of a tertiary care hospital with chief complaint of a right upper lid swelling.

Important points in history

- Prior history of sinusitis with coryzal symptoms for a week.
- She was initially treated by the GP with oral antibiotics for 10 days, but there was no improvement.
- The patient then presented acutely 1 month later with general right upper lid swelling, which was red, indurated, and tender.

Systems

- No abnormalities detected
- No prior trauma to the eye, conjunctivitis, pain on eye movement, or proptosis
- Neck and spine: no abnormalities detected
- Respiratory: no upper respiratory tract infection symptoms
- Cardiovascular: no abnormalities detected

- Gastrointestinal: no nausea, vomiting, change in bowel habit, or abdominal pain
- Extremities: warm, well-perfused peripheries
- Constitutional: no fevers, chills, rigors, or weight changes
- Musculoskeletal: no weakness or changes in sensation
- Neurological: associated frontal headache several days ago around eye but did not wake patient up at night and was not worse when leaning forward

Medical History

- Otitis media with effusion/recurrent acute otitis media 2 years ago;
- Medications: not taking any medications
- Allergies: no known drug allergies

Ocular History

- None

Family History

- No ocular disorders

Instructions to the resident

Setting:

- Mother/father of a 6-year-old girl visits out-patient department of a tertiary care hospital with chief complaint of a right upper lid swelling.
- You will take history of the patient and ask for examination findings and investigations from the facilitator whenever you require.

Instruction for the facilitator

Provide following information to the resident when asked.

External Exam/Vitals

- Vision: 20/30 OU

Pupils

- Equal, round, and reactive to light OU
- No relative afferent pupillary defect

Motility Exam

- Extraocular muscles normal

Slit-Lamp Exam

- Lids/lashes: right upper lid swelling; red, tender, and indurated
- Conjunctiva/sclera: white OU
- Cornea: clear OU
- Anterior chamber: deep and quiet
- Iris: normal
- Lens: clear

Dilated Fundus Exam

- Fundus normal
- Macula normal
- Periphery normal
- Discs 0.2 OU

Diagnostic Tests

Complete blood count: Normal.

Pediatric blood culture: Normal.

Quantitative C-reactive protein: 5 mg/L.

Imaging tests

Computed tomography, head: CT scan shows a destructive lesion superior to the right globe with destruction of right frontal bone and also the supraorbital ridge. There is intermediate-density solid material bulging into the eyelid. Bony views show a well-defined "punched out" lesion well away from the sinuses.

Magnetic resonance imaging, head: MR imaging shows a lesion mushrooming through a bony defect with peripheral enhancement and also dural enhancement. Whole-body STIR shows multiple bilateral abnormalities in the tarsal bones and also the cuboids. It is likely that the bony abnormalities in the feet reflect the patient's interest in gymnastics

Abdominal ultrasound: Abdominal ultrasound is normal with no potential primary lesion identified.

Excision biopsy and histology: There are large areas of necrosis; however, the viable tumor shows a classic Langerhans cell histiocytosis pattern with large histiocytes surrounded by eosinophils. The histiocytes are CD1a-positive."

SIMULATED PATIENT FOR 2ND YEAR RESIDENT

Instruction to the patient

During the Interview

- The interview will last approximately 15-30 minutes. Try to remain focused on the role you are playing. If possible, do not keep the written role in front of you during the interview.
- You can assist the resident by revealing some information as appropriate.
- Act naturally and react as though you are the actual patient you are portraying.

Behaviors to exhibit

- You are cooperative
- You speak Urdu well, but ask for clarification frequently, especially if medical jargon is used
- You are very concerned about seriousness of headache
- You have no problems taking the medications

Patient details

Setting

You are a 42-year-old man complaining of severe headache that began as ocular pain while playing basketball.

Important points in history

- You developed headache while playing basketball last week.
- The headache progressed as a dull ache along your forehead and left temple and later became generalized.
- Your discomfort began around his eyes.

Systems

- Skin: Negative
- Neck and spine: Negative
- Respiratory: Asthma. "When I was growing up I had to take multiple medications, but now I just use an Azmacort inhaler."
- Cardiovascular: Mild hypertension; exercise and weight loss suggested by internist.
- Gastrointestinal: Unremarkable

- Extremities: Left knee irritation
- Hematologic: Negative
- Endocrine: Negative
- Psychologic: Normal
- Neurologic: Headache. "It became so uncomfortable that after about 15 minutes I had to stop playing. Then the bright lights in the gym started bothering me. I went home and took some Tylenol. The discomfort gradually diminished, and I feel fine now."

Medical

- Ocular medications: None; contact lens wearer as teenager.
- Systemic medications: Triamcinolone acetonide inhaler; ibuprofen
- Ocular history: Photophobia. "I've noticed some glare in the gym before, but I thought it was the lighting. It never happened anywhere else."
- Medical history: Unremarkable
- Allergies: none

Family/Social

- Unremarkable

Instructions to the resident

Setting:

- A 42-year-old man has visited your department complaining of severe headache that began as ocular pain while playing basketball.
- You will take history of the patient and ask for examination findings and investigations from the facilitator whenever you require.

Instruction for the facilitator

Provide information to the resident when asked.

Clinical Exam

External

Vitals

- Temperature: 99 degrees F
- Heart rate: 45 bpm
- Blood pressure: 130/70 mm Hg
- Respiration: 14 bpm

Visual Acuity

- OD: 20/20 with correction. Manifest refraction: -0.50 sphere.
- OS: 20/25 with correction. Manifest refraction: -1.25 sphere.

Slit-Lamp Exam

- OU, dilated: The lens is clear. Pigment deposits are visible on the posterior lens capsule.

Visual Fields

Dilated Fundus Exam

- OD: The neural rim appears healthy, except superiorly, where thinning is present. Some inferotemporal sloping. Lattice degeneration.
- OS: Concentric enlargement of the optic cup, with some superior and inferior thinning. Lattice degeneration.

Pupil Exam OU: 4 mm, equal bilaterally; no RAPD.

Motility OU: Full ocular rotation in all fields of gaze.

IOP

- OD: 23 mm Hg
- OS: 30 mm Hg

Scleral transillumination results: Normal OD, abnormal OS

Confocal scanning laser ophthalmoscopy results:

- OD: Cup-disc ratio of 0.342.
- OS: Cup-disc ratio of 0.537.

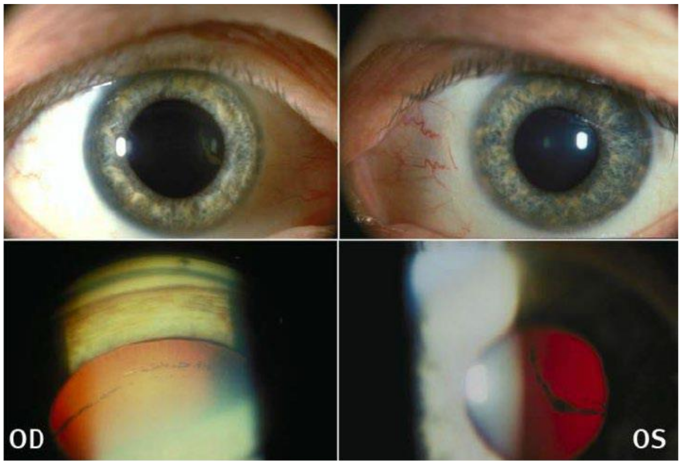
OD
OS

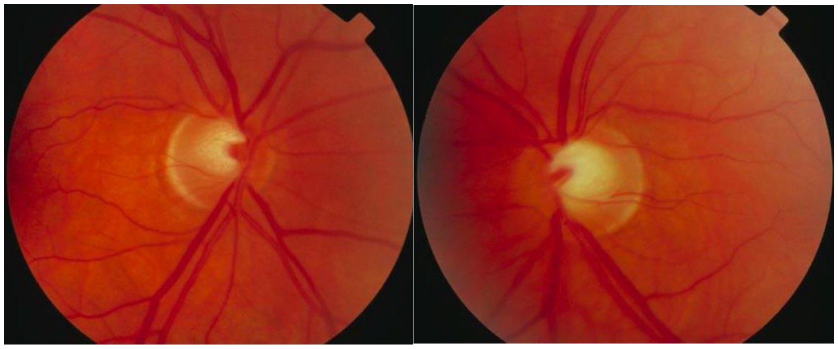

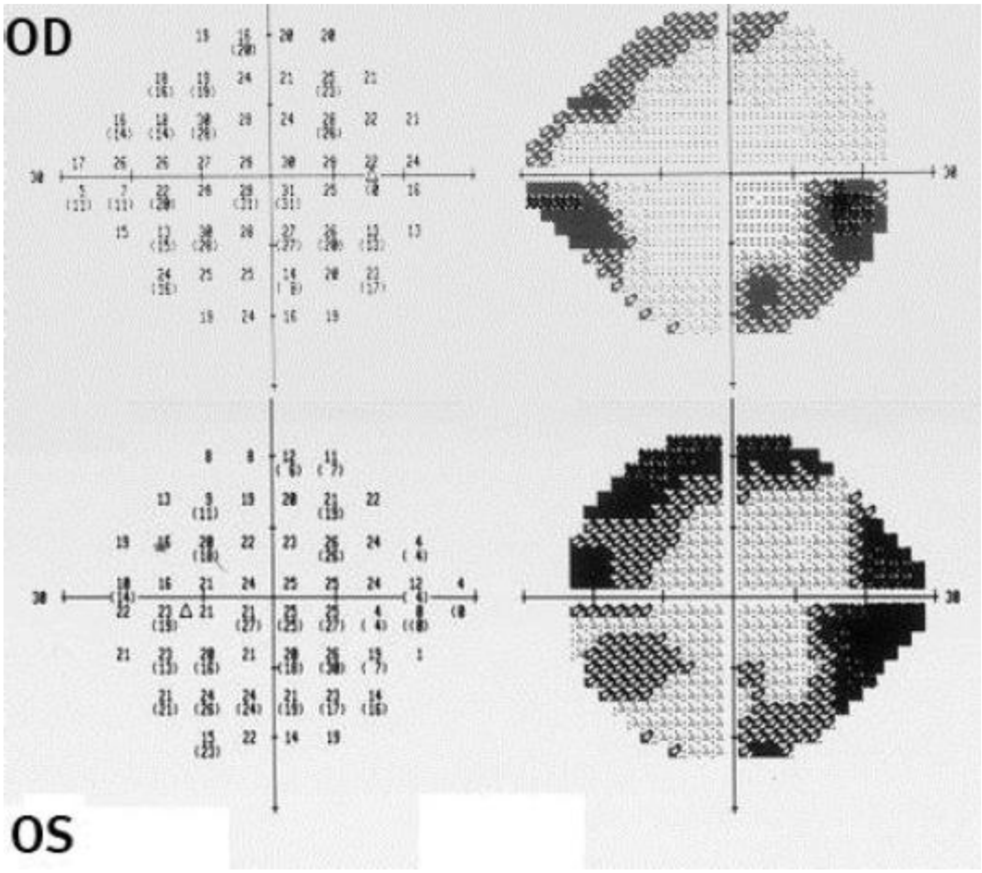
OD
OS

Ultrasound biomicroscopy results:

- OU: Iris concavity, iridolenticular contact.

Gonioscopy results: The iris insertion into the ciliary body is uniformly posterior and symmetrical in each eye. There is no evidence of peripheral anterior synechiae or neovascularization.

MRI head, orbits results: No abnormalities.

CT scan head, orbits results: No abnormalities.

Ultrasound biomicroscopy results:

- OU: Iris concavity, iridolenticular contact.

SIMULATED PATIENT FOR 3rd YEAR RESIDENT

Instruction to the patient

During the Interview

- The interview will last approximately 15-30 minutes. Try to remain focused on the role you are playing. If possible, do not keep the written role in front of you during the interview.
- You can assist the resident by revealing some information as appropriate.
- Act naturally and react as though you are the actual patient you are portraying.

Behaviors to exhibit

- You are cooperative
- You speak Urdu well, but ask for clarification frequently, especially if medical jargon is used
- You are very concerned about seriousness of the lid swelling and also concerned that the child does not allow to touch the tender lid.
- You have no problems taking the medications

Patient details

Setting

- You are mother/father of a 3-year-old boy who visits out-patient department of a tertiary care hospital with chief complaint of acute left-eye proptosis, which rapidly progressed.
- Brain and orbits CT scan confirmed the presence of a mass in the left orbit.

Systems

- No abnormalities detected
- No prior trauma to the eye, conjunctivitis, pain on eye movement, or proptosis
- Neck and spine: no abnormalities detected
- Respiratory: no upper respiratory tract infection symptoms
- Cardiovascular: no abnormalities detected
- Gastrointestinal: no nausea, vomiting, change in bowel habit, or abdominal pain
- Extremities: warm, well-perfused peripheries
- Constitutional: no fevers, chills, rigors, or weight changes
- Musculoskeletal: no weakness or changes in sensation

Medical History

- Medications: not taking any medications
- Allergies: no known drug allergies

Ocular History

- None

Family History

- No ocular disorders

Instructions to the resident

Setting:

- Mother/father of a 3-year-old boy who visits out-patient department of a tertiary care hospital with chief complaint of acute left-eye proptosis, which rapidly progressed.
- You will take history of the patient and ask for examination findings and investigations from the facilitator whenever you require.

Instruction for the facilitator

Provide information to the resident when asked.

Exam

- Vital signs: Within normal limits
- External examination:
- Right eye: Within normal limits
- Left eye: Marked proptosis with associated periorbital erythema, edema, and resistance to retropulsion
- Visual acuity: Fixes and follows with both eyes.
- Motility: Orthotropic by Hirschberg
- Head posture: normal
- Nystagmus: absent
- Visual fields: Unable to assess because of age and poor cooperation
- IOP: right: normal tactile; left: 23 mm Hg by Tonopen.
- Pupils: Both pupils brisk with no APD.
- Slit-lamp examination: Right: Within normal limits, Left: Mild chemosis and injection of the bulbar and palpebral conjunctiva
- Dilated fundus examination: Both eyes C/D 0.2 with no pallor or optic disc edema and macula, vessels, and periphery within normal limits.

Diagnostic Evaluation

CT Scan of Chest and Abdomen:

- A large mediastinal mass is noted on CT scan. Multiple solid masses in both kidneys are identified on CT of the abdomen. The abdominal aorta, inferior vena cava, stomach, liver, and spleen have a normal appearance.

MRI Scan of Orbit/Brain:

- A large, uniformly enhancing, hyperdense mass containing a plaque of ossification/calcification involving the left sphenoid wing and left lateral orbital wall is present with evidence of dural involvement of the anterior middle cranial fossa. The left globe is depressed and proptosed by the mass.

Bone Marrow Biopsy with Cytologic Studies:

- Cell morphology is most consistent with a mature B-cell acute lymphoid leukemia. The blasts are large with moderate basophilic cytoplasm with numerous vacuoles. Nucleoli are prominent.

Cytogenetic Testing:

- FISH analysis documents 8;14 translocation in cells taken from biopsy of the kidney mass.

Urine HVA and VMA:

- Within normal limits.

SIMULATED PATIENT FOR 4TH YEAR RESIDENT

Instruction to the patient

During the Interview

- The interview will last approximately 15-30 minutes. Try to remain focused on the role you are playing. If possible, do not keep the written role in front of you during the interview.
- You can assist the resident by revealing some information as appropriate.
- Act naturally and react as though you are the actual patient you are portraying.

Behaviors to exhibit

- You are cooperative
- You speak Urdu well, but ask for clarification frequently, especially if medical jargon is used
- You are very concerned about seriousness of the situation.
- You have no problems taking the medications

Patient details

Setting

You are a 67-year-old woman who presented to you after noticing that, while washing the dishes 2 hours earlier, she was unable to see out of her left eye.

Important points in history

Presentation

- Unable to see out of her left eye while washing dishes

Medical history

- Hypertension
- Hypercholesterolemia
- Rheumatoid arthritis
- Medications: Lisinopril, etanercept, and rosuvastatin

Ocular history

- Mild myopia and presbyopia
- No pain OS
- No previous floaters, flashing lights, or veils over her vision

Instructions to the resident

Setting:

- A 67-year-old woman presented to you after noticing that, while washing the dishes 2 hours earlier, she was unable to see out of her left eye.
- You will take history of the patient and ask for examination findings and investigations from the facilitator whenever you require.

Instruction for the facilitator

Provide information to the resident when asked.

Clinical Exam

- BCVA: 20/25 OD, light perception (LP) OS
- IOP: 14 mm Hg OU
- Pupils, No afferent pupillary defect
- Confrontational visual fields: Full OD, unable OS
- Extraocular motility: Full OU

Slit-lamp exam: Lids/adnexa: Normal both eyes, Conjunctiva/sclera: White and quiet OU, Cornea: Clear OU, Anterior chamber: Deep and quiet OU, Iris: Normal OU, Lens: Trace nuclear sclerosis OU

Dilated fundus exam

- Optic nerve OU: Cup/disk 0.3, mild peripapillary atrophy
- Macula OU: Flat, good foveal reflex, no cherry-red spot
- Vessels OU: Normal caliber, no tortuosity or attenuation
- Periphery OU: No holes, tears, or hemorrhages

Fluorescein angiogram

Normal filling times and no leakage seen early or late (top to bottom)

Diagnostic Tests

Lab Tests

- CBC, CRP, and ESR: Within normal limits

Imaging Tests

- Carotid dopplers: No significant narrowing on either side
- MRA of head/neck: Normal
- MRI of orbits/brain: Normal aging changes
- Transthoracic echo:
- Normal valves

Follow-Up:

- The next day, vision in her left eye is improved to 20/25 and fields are full OU.

- On hospital day 3, your patient notices a change in her peripheral vision, and she is rushed back to the eye clinic. Her vision is stable at 20/25, but she has difficulty seeing objects in her inferonasal visual field from the left eye.
